# Reports of Coding-Complete Genome Sequences of Five 2019 Novel Coronavirus (SARS-CoV-2) Strains Isolated in Bangladesh

**DOI:** 10.1128/MRA.00692-20

**Published:** 2020-07-30

**Authors:** M. Imran Khan, Kazi Nadim Hasan, Abu Sufian, M. Bayejid Hosen, Mohammed Nafiz Imtiaz Polol, M. Abdul Khaleque, M. Mizanur Rahman, M. S. M. Chowdhury, Hasan Ul Haider, Mamudul Hasan Razu, Mala Khan, Mohammad Fazle Rabbi

**Affiliations:** a DNA Solution Limited, Dhaka, Bangladesh; b Department of Biochemistry and Microbiology, School of Health and Life Sciences, North South University, Dhaka, Bangladesh; c National Forensic DNA Profiling Laboratory, Dhaka Medical College, Dhaka, Bangladesh; d Department of Plant Pathology, Sher-e-Bangla Agricultural University, Dhaka, Bangladesh; e Central Police Hospital, Dhaka, Bangladesh; f Designated Reference Institute for Chemical Measurements, BCSIR, Dhaka, Bangladesh; g Department of Soil, Water, and Environment, University of Dhaka, Dhaka, Bangladesh; h NIPRO JMI Pharma Limited, Dhaka, Bangladesh; DOE Joint Genome Institute

## Abstract

This study determined five coding-complete genome sequences of severe acute respiratory syndrome coronavirus 2 (SARS-CoV-2) strains isolated from oropharyngeal swab specimens of Bangladeshi patients who were diagnosed with coronavirus disease 2019 (COVID-19) and had no travel history.

## ANNOUNCEMENT

The recent emergence of severe acute respiratory syndrome coronavirus 2, or SARS-CoV-2, family *Coronaviridae*, genus *Betacoronavirus*, first occurred in Wuhan, Hubei Province, China, in December 2019 (1). Since then, the virus has spread globally, and the World Health Organization (WHO) declared the disease COVID-19 a pandemic on 31 January 2020 (2). Here, we report the coding-complete genomic sequences of five SARS-CoV-2 strains isolated from Bangladeshi patients with no travel history.

The oropharyngeal swab specimens were collected from Central Police Hospital in Bangladesh for three males and two females within the age range of 18 to 46 years. The same specimens were found to be positive when tested for SARS-CoV-2 using a real-time reverse transcriptase PCR (RT-PCR) kit from Sansure Biotech, Inc. (Changsha, China) and were further sequenced.

Viral RNA was extracted using a QIAamp DSP virus spin kit (Qiagen, Hilden, Germany). cDNA was prepared on the same day using a QuantiTect reverse transcription kit (Qiagen, Hilden, Germany) and used as a template for generating amplicons across the SARS-CoV-2 genome using the Ion AmpliSeq SARS-CoV-2 research panel (Thermo Fisher Scientific, Massachusetts, USA), which contains 237 pairs of unique primers in two pools specific for SARS-CoV-2. High-throughput sequence libraries were prepared using the Ion AmpliSeq library kit plus (Thermo Fisher Scientific) per the manufacturer’s protocol. The prepared libraries were quantified in a Qubit 4 fluorometer using a Qubit double-stranded DNA (dsDNA) high-sensitivity (HS) assay kit. The individual libraries were then diluted to 100 pM, pooled, and prepared for clonal amplification on the Ion OneTouch 2 instrument using the Ion 520 and 530 OT2 kits. The clonally amplified libraries were then enriched in an Ion OneTouch ES system and loaded onto an Ion 520 chip for the sequencing run. The sequencing was performed on an Ion GeneStudio S5 instrument (Thermo Fisher Scientific).

Around 7.7 million reads were generated for 5 samples. The data generated were mapped to the Wuhan virus reference sequence (GenBank accession number NC_045512) and were assembled into a consensus sequence using the built-in IRMA plugin v1.2.1.0 (3) on the Ion Reporter v5.10.0 system with default parameters. The consensus sequences were annotated using the SnpEff annotation tool v1.2.1.0 COVID-19 plugin. The assembled consensus genomic sequences were then submitted to the GenBank database. More than 98% of all the reads generated from the sequencing run were successfully mapped to the Wuhan reference genome (Table 1).

**TABLE 1:** Accession numbers and numbers of reads for individual samples and their corresponding genomic coverage

Sample name	GenBank accession no.	Length (bp)	Total no. of mapped reads	Nucleotide identity (%)	Mean depth (×)	GC content (%)
DNAS_CPH_isl_467_BGD	MT566434	29,642	1,949,315	99.97	12,005	38
DNAS_CPH_isl_471_BGD	MT566435	29,833	1,767,571	99.96	10,512	38
DNAS_CPH_isl_427_BGD	MT566436	28,829	1,917,849	99.96	12,065	38
DNAS_CPH_isl_466_BGD	MT566437	29,828	298,009	99.98	1,813	38
DNAS_CPH_isl_436_BGD	MT566438	29,706	1,717,970	99.97	10,451	38

Five SARS-CoV-2 sequences from the study were compared to existing genomes using online NCBI BLAST (https://blast.ncbi.nlm.nih.gov/Blast.cgi). Comparison of our five isolate genomes (GenBank accession numbers MT566434, MT566435, MT566436, MT566437, and MT566438) revealed 99.97%, 99.96%, 99.96%, 99.98%, and 99.97% nucleotide identities, respectively, with the Wuhan virus reference sequence (NC_045512).

### Data availability.

These sequences have been deposited in GenBank under the accession numbers MT566434, MT566435, MT566436, MT566437, and MT566438 and at the GISAID EpiCoV newly emerging coronavirus SARS-CoV-2 platform under the identifier EPI_ISL_445213-17. The accession numbers for the Ion Torrent sequence raw reads in the NCBI Sequence Read Archive (SRA) are PRJNA643654 (BioProject) and SRR12132975 to SRR12132979 (SRA). The BioSample numbers are SAMN15422957 (DNAS_CPH_436), SAMN15422958 (DNAS_CPH_466), SAMN15422959 (DNAS_CPH_427), SAMN15422960 (DNAS_CPH_471), and SAMN15422961 (DNAS_CPH_467).
